# Advance Care Plans for Sexual Minority Older Adults: Disparities Across Lesbian, Gay, and Bisexual Individuals

**DOI:** 10.1093/geroni/igaa057.3171

**Published:** 2020-12-16

**Authors:** Britney Wardecker, Jes Matsick

**Affiliations:** The Pennsylvania State University, University Park, Pennsylvania, United States

## Abstract

To ensure preference-based care, health providers advise individuals to establish advance care plans (e.g., power of attorney, valid will; NIA, 2018). Though important for all individuals to prepare for declining health, it is critical for bisexual older adults to establish plans given they evidence more health problems compared to heterosexual, lesbian, and gay counterparts (Fredriksen-Goldsen et al., 2016). To assess factors related to having advance care plans, we used data from sexual minority older adults (n=158) in the Health and Retirement Study (2016). Bisexual individuals reported a lower number of valid wills than lesbian and gay participants, t(152)=3.80, p<.001. This finding is particularly alarming given bisexual people are the numeric majority of sexual minorities and experience elevated health problems in older age. We will discuss mechanisms of disparities, the need for research on end-of-life care for sexual minorities, and implications for improving healthcare providers role in facilitating care plans.

